# Evaluation of the Release Kinetics of a Pharmacologically Active Substance from Model Intra-Articular Implants Replacing the Cruciate Ligaments of the Knee

**DOI:** 10.3390/ma12081202

**Published:** 2019-04-12

**Authors:** Dorota Wójcik-Pastuszka, Justyna Krzak, Bartosz Macikowski, Ryszard Berkowski, Bogdan Osiński, Witold Musiał

**Affiliations:** 1 Department of Physical Chemistry, Faculty of Pharmacy, Wroclaw Medical University, ul. Borowska 211A, 55-556 Wroclaw, Poland; dorota.wojcik-pastuszka@umed.wroc.pl (D.W.-P.); bartosz.macikowski@gmail.com (B.M.); ryszard.berkowski@umed.wroc.pl (R.B.); 2 Department of Mechanics, Materials Science and Engineering, Faculty of Mechanical Engineering, Wroclaw University of Science and Technology, ul. Smoluchowskiego 25, 50-370 Wrocław, Poland; justyna.krzak@pwr.edu.pl; 3 Department and Clinic of Surgery, Faculty of Veterinary Medicine, Wroclaw University of Environmental and Life Sciences, pl. Grunwaldzki 51, 50-366 Wroclaw, Poland; vetscidr@gmail.com

**Keywords:** betamethasone, implants, ligaments, drug release, kinetics

## Abstract

Implants are readily applied as a convenient method of therapy. There is great interest in the prolonged release of active substances from implants. The objective of this work was to evaluate the dissolution kinetics of steroidal anti-inflammatory preparation (SAP) released from novel implants, and to test the influence of the technology on SAP release kinetics. The proposed long-acting preparations may overcome difficulties resulting from repeated injections and often visits to ambulatory clinic, as the stabilizing function of the artificial ligament would be enriched with pharmacological activity. The potential advantages provided by the new coatings of knee implants include the continuous, sustained, and prolonged release of an active substance. The study was carried out using a modified United States Pharmacopoeia (USP) apparatus 4. The amount of SAP was measured spectroscopically. It was revealed that the transport of the drug was mainly a diffusion process. The drug release kinetics was analyzed using zero-, first-, and second-order kinetics as well as Korsmeyer-Peppas, Higuchi, and Hixon-Crowell models. The highest values of the release rate constants were k_0_ = (7.49 ± 0.05) × 10^−5^ mg × min^−1^, k_1_ = (6.93 ± 0.05) × 10^−6^ min^−1^, and k_2_ = (7.70 ± 0.05) × 10^−7^ mg^−1^ × min^−1^ as calculated according to zero-, first-, and second-order kinetics equations, respectively. The values of the rate constants obtained for the slowest process were k_0_ = (3.63 ± 0.06) × 10^−5^ mg × min^−1^, k_1_ = (2.50 ± 0.03) × 10^−6^ min^−1^, and k_2_ = (2.80 ± 0.03) × 10^−7^ mg^−1^ × min^−1^. They may suggest the possibility of sustained release of betamethasone from the system. Due to the statistical analysis, differences were observed between most of the studied implants. Incubation, temperature, time of stabilization of layers, and the method of SAP deposition on the matrix affected the drug release.

## 1. Introduction

The optimal mechanical properties of musculoskeletal joints are ensured by cartilage and synovial fluid. The failure of these tissues as a result of various arthritis forms leads to the development of diseases [1,2]. For the treatment of osteoarthritis (OA), non-steroidal and anti-inflammatory drugs (NSAIDs) are most commonly used [3,4]. However, the long-term use of painkillers and anti-inflammatory drugs can reduce the symptoms of illness but at the same time can accelerate the destruction of joints. Moreover, it was found that patients receiving NSAIDs may encounter upper gastrointestinal side effects [5,6]. Treating the disease itself, and not just its symptoms, increases the recovery chance. Pentosan polysulfate and polysulfated glycosaminoglycan seem to act on the pathologies responsible for its primary causes [7,8,9,10,11,12,13]. Corticosteroid medications are used to treat OA as well [14]. For example, the intra-articular administration of betamethasone is effective in controlling OA symptoms [15,16]. However, these injections may result in joint infections and are not recommended for patients with diabetes mellitus [16]. Another type of OA therapy are invasive interventions, e.g., intra-articular injections and surgical treatments [17].

The strategy for the management of joint pain promotes the formation of artificial ligaments with biocompatible or bioactive coatings [18]. Müller et al. [19] proposed a polypropylene mesh as a substitute for the ligament. These implants enabled early recovery and limb mobility. The accurate strength of the implant resulted in minimalization of external immobilization.

Conventional pharmacological agents have relatively short durations of action, what results in vulnerability to non-adherence. To overcome these difficulties, long-acting preparations have been proposed such as long-term implants with active substances. The benefits of such products include prolonged effect obtained by a drug reservoir and the continuous, sustained release of an active substance [20]. Long-acting therapy can replace practices based on drug injections or recipience of oral forms [21]. It provides a clinically attractive option for long-term therapy in patients seeking fewer office visits and fewer repeated injections [22]. The study of risperidone subcutaneous implants indicated that this type of delivery system provides consistent therapeutic blood levels. Moreover, benefits include improved medication adherence, the ability to withdraw the medication if needed due to treatment-emergent adverse effects, fewer relapses, and improved efficacy [23]. Zhang et al. [24] used a long-acting intravitreal implant containing ligustrazine for the treatment of proliferative vitreoretinopathy. The above presented study found that the in vitro drug release from the system fits to zero-order kinetics. Application of the evaluated implants significantly reduced the development of the disease. Jinagal et al. [25] studied the safety and advantages of intravitreal implants containing dexamethasone in patients with juvenile idiopathic arthritis associated uveitis. Children were observed for a period of 6 months., Ocular inflammation, intraocular pressure, best-corrected visual acuity, and worsening of uveitis were assessed. No changes were noticed at various follow-up visits. It was concluded that this therapy is safe and useful in preventing and treatment of the postoperative inflammation in children. Bishop et al. [26] proposed vancomycin as an additive to orthopedic bone cement in treatment of infections after total hip or knee arthroplasty. The drug can play a role a medium against infection. The antibiotic was incorporated into bone cement to ensure the drug distribution at the implant site. It can elute out of the cement at a controlled concentration that is active against common organisms. It was revealed that vancomycin was released for 8 days from the cement at therapeutic levels. This period was sufficient to eliminate *S. aureus*; however the applied dose of the drug was insufficient against *S. epidermidis*.

The aim of this work was evaluation of the kinetics of the release of a steroidal anti-inflammatory preparation (SAP) from implants that are replacing the cruciate ligaments of the knee. The influence of a deposition technique on the kinetic parameters of the drug release from the polymeric system was studied.

The research of the drug release kinetics from a preparation may elucidate molecular patterns of dissolution of the active ingredient, and refine its stability properties. The parameters obtained by fitting various kinetic models to the experimental data play a significant role in understanding the release mechanism.

## 2. Materials and Methods

Implants were obtained from Wroclaw University of Science and Technology. In brief, SAP was applied directly to a polyester cord with a length of 5 cm or incorporated into a silica layer, which was then applied to the polymer. The procedure for the synthesis of samples was as follows. The substrates used for coating applications were polyester cords (PC), applied for the surgical replacement of knee ligaments. These prostheses were made of the polyester yarn DALLOS produced by Tricomed, Łódź, Poland. Silica coatings (SiO_2_) were synthesized by the sol-gel method, which was based on the hydrolysis and condensation reaction of an oxide precursor [27]. In our experiment, reactions used tetraethoxysilane and diethoxydimethylsilane as precursors and ethanol as a solvent. Sol-gel synthesis was based on acid hydrolysis. The coating on the polyester substrate was obtained using an ultrasonic bath. For sample activation, we used a 1 mL suspension of SAP that contained 6.43 mg betamethasone dipropionate and 2.63 mg betamethasone sodium phosphate under the commercialized name Diprophos (Schering-Plough Labo N.V., Heist-op-den-Berg, Belgium). Samples were activated according to the methods presented in Table 1. The activation of the considered samples differed in the manner of betamethasone interaction with the SiO_2_ matrix and in the temperature of stabilization. By functionalization at the synthesis stage, the entire volume of coatings was activated, whereas by incubation.

Sodium hydroxide and anhydrous potassium dihydrogen phosphate were delivered from Chempur (Poland, Piekary Śląskie, Poland). All chemicals were pharmaceutical grade and used without further purification. Phosphate buffer solution, pH 7.4, was prepared according to the European Pharmacopoeia [28]. SAP was diluted in phosphate buffer solution and effected in the concentration of the drug ca. 0.03624 mg/mL. The UV-Vis spectrum (Jasco V-530, Tokyo, Japan) of the substance was recorded from 200 to 900 nm, with a scanning speed of 500 nm per min at room temperature. The characteristic absorption band was observed at 240 nm. At this wavelength, the absorbance of 5 various concentrations of SAP ranging from 0.00604–0.01812 mg/mL were read to prepare the calibration curve. The composition of SAP was: betamethasone sodium phosphate and betamethasone dipropionate, disodium phosphate dehydrate, disodium phosphate anhydrous, sodium chloride, disodium edetate, polysorbate 80, benzyl alcohol, methyl parahydroxybenzoate, propyl parahydroxybenzoate, sodium carboxymethylcellulose, macrogol, hydrochloric acid. The measured spectrum of SAP is presented in Graphical abstract. Based on literature data the observed band at 240 nm has been assigned to both betamethasone sodium phosphate and betamethasone dipropionate [28,29,30]. No additional peaks in the wavelength range 200–900 nm at the used conditions were observed. This observation suggested that excipients do not present signal in the above-mentioned region. The spectrum of the same mixture stored for 24 h was the same as the spectrum of fresh mixture. It indicated the lack of disturbances from any decomposition products. Unlike conventional dosage forms, there is a lack of standard pharmacopoeial or other regulatory tests dedicated for parenteral, controlled-release drug products in the form of implanted ligaments. In particular, a method for the in vitro release of a drug from implants was not found. Burgess et al. [31], and Siewert et al. [32] proposed combined methods or their modification for the study of the in vitro dissolution of a drug from novel or special formulations. The validation of USP apparatus 4 used for the in vitro drug release study from microspheres revealed that the dissolution of the drug was not affected by the flow rate in the assessed flow range, the number of microspheres, or the cell size. However, slight changes in the temperature affected the differences in drug dissolution profiles [33].

**Figure 1:**
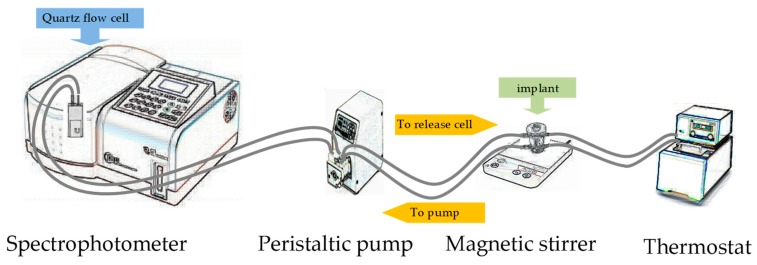
A schematic representation for the release apparatus.

In the present work, the release study was carried out using a modified USP apparatus 4—the flow-through cell equipment (Laboratory Glass Apparatus, Inc., Berkeley, CA, USA). The acceptor fluid used in the experiment was phosphate buffer solution, pH 7.4 (USP 32), at temperature of 37 °C. The temperature was controlled using a thermostat (Prüfgeräte-Werk, B2 E10, Medingen, Germany). The drug content in the samples was determined by UV measurements (JASCO V-530, Tokyo, Japan), reading the absorbance every 2 min at a wavelength of 240 nm. A flow rate of 5 mL/min was established by employing a peristaltic pump (PS-16 Sipper Pump, PG Instruments Limited, Leicestershire, UK), and the rotation speed of the fluid was 100 rpm (magnetic stirrer, 2 mag magnetic emotion, Munich, Germany). Each experiment was conducted in triplicate. The schematic representation for the release apparatus is presented in Figure 1.

It was found that the drug release from implants depends on the test method as well as the media used in the experiment. Differences between the release profiles obtained using various dissolution tests were observed. These results suggest the necessity of knowledge about the underlying in vivo processes and the need to translate this research to the in vitro test systems [34].

The obtained release profiles were analyzed with zero-, first-, and second-order kinetics as well as Korsmeyer-Peppas [35,36] Higuchi [37] and Hixon-Crowell models [38].

The zero-order kinetics model is as follows:

(1) $$m_{t}=m_{b}+k_{0}t$$

where m_t_ is the amount of the drug released over time t, m_b_ is the amount of the drug in solution before release (usually it is 0), and k_0_ is the zero-order release rate constant.

The first-order kinetics model is as follows:

(2) $$\ln\left(m_{0}-m_{t}\right)=\ln\left(m_{0}\right)-k_{1}t$$

where m_0_ is the amount of the drug in the formulation before dissolution, and k_1_ is the first-order release rate constant.

The second-order kinetics model is as follows:

(3) $$\frac{1}{\left(m_{0}-m_{t}\right)}=\frac{1}{m_{0}}-k_{2}t$$

where k_2_ is the second-order rate constant.

The Korsmeyer-Peppas model [35,36,39] is as follows:

(4) $$\log\left(\frac{m_{t}}{m_{\infty }}\right)=\log k_{K–P}+n\log t$$

where m_∞_ is the amount of the drug released after an infinitive time (in this research after 24 h), k_K–P_ is the Korsmeyer-Peppas rate constant, and n is the parameter indicative of the drug release mechanism.

The Higuchi model [37] is as follows:

(5) $$m_{t}=k_{H}t^{0.5}$$

where k_H_ is the Higuchi rate constant.

The Hixon-Crowell model [38] is as follows:

(6) $$m_{0}{}^{1/3}-m_{\mathrm{left}}{}^{1/3}=k_{H–C}t$$

where m_left_ is the amount of drug left in the formulation over time t, and k_H–C_ is the Hixon-Crowell rate constant.

Moreover, the difference factor, f_1_, was calculated to compare the dissolution profiles [40,41]:

(7) $$f_{1}=\frac{\sum_{t=1}^{n}\left|R_{t}-T_{t}\right|}{\sum_{t=1}^{n}R_{t}}\times 100$$

where n is the number of time points, R_t_ is the dissolution value of the reference batch at a given time (t), and T_t_ is the dissolution value of the test batch at time t.

Linear regression analysis based on the least-squares regression method was employed to study the linearity of the kinetic models. Comparing the standard deviation (SD) and the correlation coefficient, R^2^, allowed us to choose the kinetic model that describes the observed processes well. Statistical analysis using analysis of variance (ANOVA) with Tukey’s test and Student’s *t*-test were used to assess the differences between the obtained release profiles. A statistically significant difference was indicated when *p* < 0.05 [42].

## 3. Results and Discussion

### 3.1. Mechanism of the Drug Release

The transport of drugs from pharmaceutical systems involves various physical and chemical rules, what results in difficulties in ascribing proper mathematical model to the occurring processes. Zero-order kinetics may be used if the pharmaceutical dosage form does not disaggregate and release the drug slowly. First-order model may describe the early stage of dissolution of a poorly water-soluble drug embedded in water-soluble matrix. Higuchi equation is based on several assumptions: the initial concentration of the drug in the formulation is higher than the drug solubility; the drug spreads only in one dimension; the substance particle are smaller than the size of a carrier; swelling of the system and its dissolution is insignificant; the drug diffusivity does not change; sink condition are achieved. Hixon-Crowell model may be applied when the drug is released on parallel planes of the drug form surface, e.g., in the case of tablet, when the size reduces proportionally and the geometrical shape stay constant [43,44]. Siepmann and Peppas [39] observed that the geometry of the system, the amount of the drug as well as water-solubility of the drug can exclude some of the models. In the present work several equations were used to fit the experimental data to the theoretical curve. Zero-, first-, and second-order kinetics were employed to describe the release of SAP from all implants studied and the results are listed in Table 2. However, in the case of implants E, F, and G, it was hard to fit the release data to the Higuchi model. The Korsmeyer-Peppas model was unsuitable for describing the release of the drug from implant F, and the Hixon-Crowell model was inappropriate to reflect the dissolution of SAP from implants A and C. The values of the correlation coefficient R^2^ indicate which kinetic model is appropriate for the release of the drug. Gouda et al. [43] obtained the value of R^2^ between 0.933 and 0.9157, and discussed that the release kinetics do not perfectly follow the used model, although it is gently approaching. This observation suggests that the drug was transported from the studied systems in various ways. A regression coefficient value, R^2^, close to 1 indicated the model fitting the release mechanism. For implants A–D, the best kinetic model was the Higuchi equation, as the plots showed high linearity, with R^2^ values in the range 0.9872–0.9962, suggesting mainly the diffusion process [45]. These results were consistent with results obtained from Korsmeyer-Peppas model analysis for systems A and C. According to this model, the drug was transported via Fickian diffusion when the value of the parameter n was below or equal to 0.5, while the n value between 0.5 and 1.0 indicated anomalous transport [35,39]. In the case of implants B and D, the n values were slightly above 0.5, namely, 0.582 ± 0.004 and 0.579 ± 0.005, suggesting a coupling of diffusion and erosion mechanisms, anomalous diffusion, and may indicate that betamethasone release from implants B and D was controlled by more than one process. However, Antesh et al. [46] obtained n values between 0.4225 and 0.7309 in their study and interpreted the observed transport of the active substance was controlled by diffusion. In the present work, fitting the data to the Higuchi equation together with n values slightly above 0.5, may suggest diffusion-controlled drug release. In the case of implant E, the n value was 0.46 ± 0.01, suggesting diffusion of the drug. However, the regression coefficient, R^2^, of implant E in the Korsmeyer-Peppas model has the lowest value (0.8113) in comparison to the R^2^ value obtained for the rest of implants. The highest R^2^ value in this kinetic model (0.9722) was obtained for the G system and corresponded to the highest n value (0.75 ± 0.01) among all implants studied. This value of n above 0.5, together with the non-adherence of the experimental data to the Higuchi model, may be regarded as an indicator of the anomalous transport of the drug. The data derived from the dissolution of betamethasone from implants F were unsuitable for analysis with the Higuchi and Korsmeyer-Peppas models.

The dissolution data were also plotted according to the Hixon-Crowell model. The possibility of describing the release of betamethasone from implants B and D–G using this model may indicate that the change in the surface area and in the diameter of particles during the dissolution process affected the drug release. The difficulties with describing the dissolution process of betamethasone from implants A and C, using the Hixon-Crowell equation, may indicate that changes of surface area and of diameter slightly influenced the release.

It could be concluded that diffusion was the dominant mechanism of betamethasone release from implants A–E. The temperature and time of stabilization of layers and the incubation of implants did not influence the mechanism of betamethasone transport. These results contrasted with results obtained for implants F–G. The release of the drug from these implants was best expressed by first-order kinetics, and betamethasone was transported via non-Fickian diffusion. This could be a result of more silica layers that contained the drug applied on polyester than in implants A–E (Table 1). SAP molecules must overcome the silica layers to reach the surface of the implant. This process was not simple diffusion, which was confirmed by the value of parameter n above 0.5 in the case of the G system. The release of betamethasone from implants B and D–G was connected to the change in the surface area and diameter of particles along with the progressive dissolution process.

### 3.2. Dissolution Study

**Figure 2:**
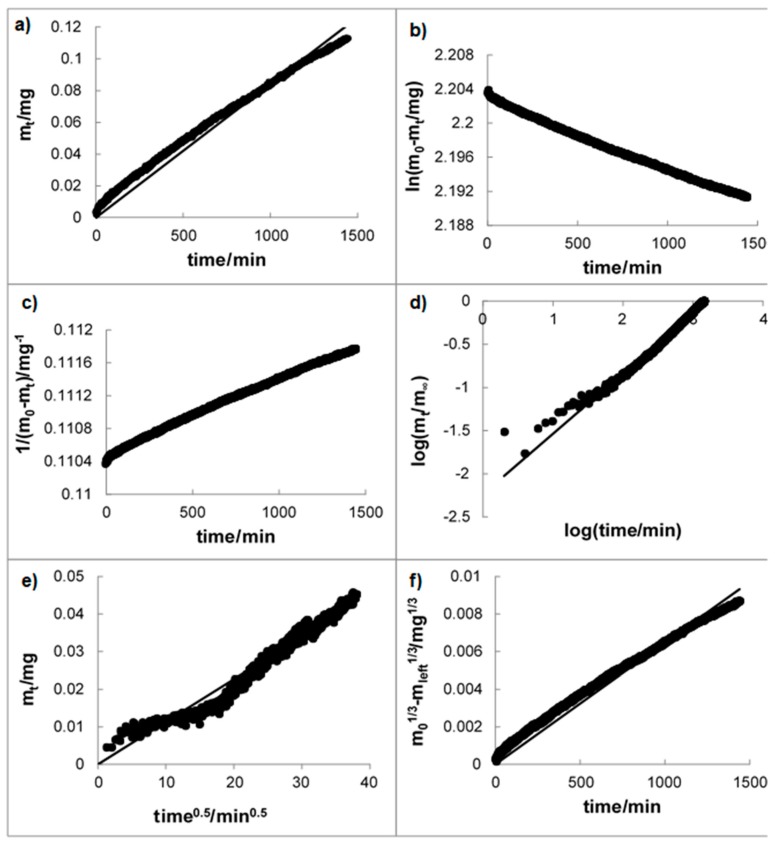
Release kinetics of SAP from implant B according to (**a**) zero-order kinetics, (**b**) first-order kinetics, (**c**) second-order kinetics, (**d**) the Korsmeyer-Peppas model, (**e**) the Higuchi model, and (**f**) the Hixon-Crowell model; experimental data ●, solid line: linear regression.

**Figure 3:**
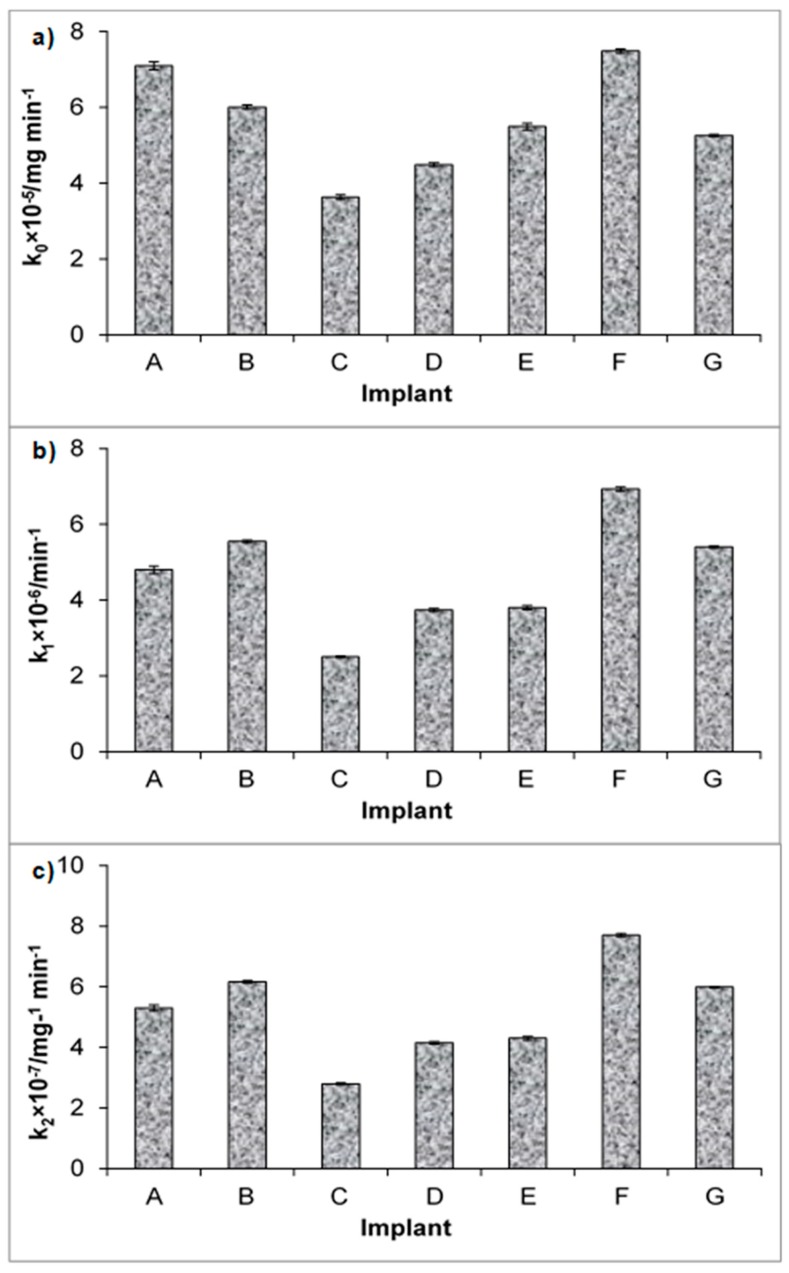
Comparison between the values of the release rate constants obtained by fitting the experimental data to: (**a**) zero-order kinetics, (**b**) first-order kinetics, (**c**) second-order kinetics.

The kinetic release parameters and regression coefficients calculated from various kinetic models, such as zero-, first-, and second-order kinetics, as well as the Higuchi [37], Korsmeyer-Peppas [35,36,39] and Hixon-Crowell [38] models, are listed in Table 2. Exemplary fitting of release models to experimental data obtained from the dissolution of SAP from implant B is shown in Figure 2. In Figure 2a, the application of zero-order kinetics is presented. This model describes the drug release rate from pharmaceutical dosage forms independent of the drug concentration in contrast to the first-order kinetics, where the rate was concentration dependent, meaning the greater the concentration, the faster the process. The first-order model is illustrated in Figure 2b. The second-order kinetics, when the rate depends on the concentration of the drug in the second power, is presented in Figure 2c. The comparison of the rate constants derived employing zero-order, first-order, and second-order kinetics for all implants studied is attached in Figure 3. It should be noted that the highest value of the rate constant was achieved for implant F, and the lowest value was obtained for implant C, reflecting the release rates for F and C, respectively. According to these observations, implant C was able to prolong and control betamethasone release. Based on zero-order kinetics that describe the release of SAP from implant C well, the half-release time is 133,064 ± 1881 min, which corresponds to a period of approximately three months (92 days).

The half- release time in implant F, calculated using first-order kinetics, which expressed the release of SAP well, was 102,166 ± 763 min, corresponding to a period of approximately 72 days. It is worth mentioning that both implants C and F were incubated, suggesting that this process does not influence the kinetics of SAP release from these implants. The difference in kinetic parameters may be attributed to the temperature and time of the silica layer stabilization. Moreover, these discrepancies may occur due to the methods of SAP application on the polymeric system. Comparing the kinetic parameters obtained from tested models (Table 2, Figure 3), the values were different for each implant.

For the evaluation of in vitro release differences, a simple model-independent approach, based on the calculation of a difference factor f_1_ [41,42], was employed. The results of the calculations are listed in Table 3. The differences in the release studies of the drug were noticed in each instance, with f_1_ values below 15. These results demonstrated the inconsistency in the release of SAP from all implants studied, except for comparisons of systems: E and D, B and G, A and F. In these comparisons, the differences were not observed.

In general, it could be concluded that the incubation, the method of SAP deposition and the temperature and time of silica layer stabilization influence the kinetics of drug release.

### 3.3. Statistical Analysis

To confirm the differences in the release of SAP from the implants tested, statistical analysis by ANOVA, together with Tukey’s test, was performed [41]. The obtained value of *F* was 245 and was higher than the critical value of *F* = 2.22. The SAP dissolution profiles were significantly different. All possible pairs of SAP dissolution profiles were compared using Tukey’s test to determine exactly where are the differences, which indicated that specific groups compared with each other were different. The calculated value of the honest significant difference was 0.032. The data coming from comparisons of the individual groups were collected in Table 4 and were predominantly higher than 0.032.

There were significant differences between the SAP dissolution profiles from all implants tested, apart from E and G, as well as A and F. Only in these two cases the value of NIR was below 0.032. These results are consistent with the results obtained from Student’s *t*-test analysis. According to the data presented in Table 5, differences in dissolution profiles exist between all dissolution profiles of SAP, except two pairs: E and G, as well as A and F. In these cases, the value of *t* is lower than the critical value of 1.96, reflecting statistical similarity of the release profiles.

## 4. Conclusions

In conclusion, diffusion was the dominant mechanism of betamethasone release from implants A–E. The temperature and time of stabilization of the layers as well as incubation of implants did not change the transport mechanism of betamethasone. In the case of implants F–G, the drug dissolution was expressed by first-order kinetics, and betamethasone was transported via non-Fickian diffusion. The dissolution of the drug from the assessed implants could be described by zero-, first-, and second-order kinetic equations. The release rate constants obtained from these models had the highest value for implants F and the lowest value for implants C. This model-independent analysis confirmed the variability of the release of the drug in most cases between the different implants.

## Figures and Tables

**Table 1:** Conditions of the preparation of evaluated implants.

Implant Type	SAP Activation Procedure				Temperature and Time of Coating Stabilization
	Polymer Substrate	Coating	Implementation of SAP during Synthesis of the Coating	Implementation of SAP to Final Material	
**A**	Polyester	-	-	SAP	35 °C; 10 h
**B**	Polyester	-	-	SAP	125 °C; 5 h
**C**	Polyester	SiO_2_	-	SAP	125 °C;5 h
**D**	Polyester	SiO_2_	SAP	-	35 °C; 10 h
**E**	Polyester	SiO_2_	SAP	-	125 °C; 5 h
**F**	Polyester	SiO_2_	SAP	SAP	35 °C; 10 h
**G**	Polyester	SiO_2_	SAP	SAP	125 °C; 5 h

SAP—steroidal anti-inflammatory preparation with betamethasone.

**Table 2:** The obtained kinetic parameters.

Kinetic Model	Kinetic Parameters	A	B	C	D	E	F	G
Z–O	k_0_ × 10^5^/mg min^−1^	7.1 ± 0.1	6.01 ± 0.05	3.63 ± 0.06	4.49 ± 0.05	5.49 ± 0.09	7.49 ± 0.05	5.26 ± 0.03
	R^2^	0.9451	0.9830	0.9568	0.9771	0.9614	0.9880	0.9978
	t_0.5_/min	69,088 ± 1136	89,563 ± 949	133,064 ± 1881	101,189 ± 1142	91,966 ± 1571	61,461 ± 433	92,293 ± 438
F–O	k_1_ × 10^6^/min^−1^	4.8 ± 0.1	5.55 ± 0.04	2.5 ± 0.03	3.74 ± 0.04	3.8 ± 0.05	6.93 ± 0.05	5.4 ± 0.02
	R^2^	0.9216	0.9841	0.9802	0.9799	0.9419	0.9896	0.9986
	t_0.5_/min	154,398 ± 2836	161,737 ± 1708	275,687 ± 2926	185,248 ± 1920	184,738 ± 3086	102,166 ± 763	135,310 ± 456
S–O	k_2_ × 10^7^/mg^−1^ min^−1^	5.3 ± 0.1	6.16 ± 0.04	2.8 ± 0.03	4.15 ± 0.04	4.3 ± 0.06	7.70 ± 0.05	5.99 ± 0.02
	R^2^	0.9218	0.9842	0.9802	0.9801	0.9420	0.9895	0.9981
	t_0.5_/min	221,412 ± 4063	232,403 ± 2445	396,449 ± 4212	266,269 ± 2746	265,355 ± 4425	146,461 ± 1099	194,543 ± 654
H	k_H_ × 10^3^/mg × min^−1/2^	2.27 ± 0.02	1.87 ± 0.02	1.15 ± 0.009	1.406 ± 0.006	-	-	——
	R^2^	0.9872	0.9898	0.9878	0.9962	-	-	——
	t_0.5_/min	334 ± 3	438 ± 3	648.3 ± 5.5	495 ± 2	-	-	——
K–P	k_K–P_ × 10^3^/min^−n^	68.7 ± 3.7	18.2 ± 1.2	71.0 ± 4.0	16.6 ± 0.7	56.2 ± 4.5	-	4.6 ± 0.3
	n	0.368 ± 0.006	0.582 ± 0.004	0.351 ± 0.007	0.579 ± 0.005	0.46 ± 0.01	-	0.75 ± 0.01
	R^2^	0.8967	0.9508	0.8744	0.9667	0.8113	-	0.9722
	t_0.5_/min	332.0 ± 58.6	493 ± 47	394 ± 77	461 ± 41	399 ± 57	-	564 ± 49
H–C	k_H–C_ × 10^6^/mg^1/3^min^−1^	-	4.63 ± 0.04	-	3.58 ± 0.04	3.8 ± 0.05	6.07 ± 0.04	3.25 ± 0.01
	R^2^	-	0.9831	-	0.9743	0.9615	0.9922	0.9989
	t_0.5_/min	-	110,648 ± 1170	-	120,126 ± 1448	54,503 ± 720	71,870 ± 460	132,865 ± 372
Best fit		H	H	H	H	H–C, Z–O	F–O	F–O

Z–O—zero order; F–O—first order; S–O—second order; H—Higuchi model; K–P—Korsmeyer-Peppas model. H–C—Hixon-Crowell model.

**Table 3:** The obtained values of the difference factor f_1_.

Implant	A	B	C	D	E	F
**A**	-	-	-	-	-	-
**B**	27	-	-	-	-	-
**C**	50	37	-	-	-	-
**D**	40	23	22.5	-	-	-
**E**	32	17	37	13	-	-
**F**	14	24	49	38.5	31	-
**G**	31	12	47	22	18	29

**Table 4:** The values of near-infrared (NIR)-test Tukey.

Implant	A	B	C	D	E	F
**A**	-	-	-	-	-	-
**B**	0.139	-	-	-	-	-
**C**	0.325	0.186	-	-	-	-
**D**	0.259	0.120	0.066	-	-	-
**E**	0.207	0.068	0.118	0.052	-	-
**F**	0.017	0.122	0.308	0.242	0.190	-
**G**	0.199	0.061	0.125	0.059	0.007	0.183

**Table 5:** The calculated values of statistic *t*.

Implant	A	B	C	D	E	F
**A**	-	-	-	-	-	-
**B**	12.21	-	-	-	-	-
**C**	37.95	19.36	-	-	-	-
**D**	27.22	11.42	9.23	-	-	-
**E**	21.51	6.35	16.26	6.25	-	-
**F**	1.19	8.98	26.81	19.83	15.47	-
**G**	16.75	4.71	12.44	5.45	0.65	12.93
